# The Waterlow score for risk assessment in surgical patients

**DOI:** 10.1308/003588413X13511609954770

**Published:** 2013-01

**Authors:** CC Thorn, M Smith, O Aziz, TC Holme

**Affiliations:** East and North Hertfordshire NHS Trust,UK

**Keywords:** General surgery, Risk assessment, Mortality, Morbidity

## Abstract

**Introduction:**

Perioperative scoring systems aim to predict outcome following surgery and are used in preoperative counselling to guide management and to facilitate internal or external audit. The Waterlow score is used prospectively in many UK hospitals to stratify the risk of decubitus ulcer development. The primary aim of this study was to assess the potential value of this existing scoring system in the prediction of mortality and morbidity in a general surgical and vascular cohort.

**Methods:**

A total of 101 consecutive moderate to high risk emergency and elective surgical patients were identified through a single institution database. The preoperative Waterlow score and outcome data pertaining to that admission were collected. The discriminatory power of the Waterlow score was compared against that of the American Society of Anesthesiologists (ASA) grade and the Portsmouth Physiological and Operative Severity Score for the enUmeration of Mortality and morbidity (P-POSSUM).

**Results:**

The inpatient mortality rate was 17% and the 30-day morbidity rate was 29%. A statistically significant association was demonstrated between the preoperative Waterlow score and inpatient mortality (*p*<0.0001) and 30-day morbidity (*p*=0.0002). Using a threshold Waterlow score of 20 to dichotomise risk, accuracies of 0.84 and 0.76 for prediction of mortality and morbidity were demonstrated. In comparison with P-POSSUM, the preoperative Waterlow score performed well on receiver operating characteristic analysis. With respect to mortality, the area under the curve was 0.81 (0.80–0.85) and for morbidity it was 0.72 (0.69–0.76). The ASA grade achieved a similar level of discrimination.

**Conclusions:**

The Waterlow score is collected routinely by nursing staff in many hospitals and might therefore be an attractive means of predicting postoperative morbidity and mortality. It might also function to stratify perioperative risk for comparison of surgical outcome data. A prospective study comparing these risk prediction scores is required to support these findings.

The measurement of hospital and surgeon performance has received much attention and has become widespread in UK hospitals, performed in both retrospective and prospective settings. The Charlson co-morbidity index is a method of risk stratification that is used to compare surgical outcomes between UK hospitals (hospital standardised mortality ratio).^1^ The required variables are derived from Hospital Episode Statistics (HES) data, which hospital trusts are obliged to submit on a quarterly basis. This index was derived originally from a population of medical patients and has been adapted for contemporary medical practice and validated to predict the ten-year mortality for patients, relating to their co-morbidity.^2^ The capture of relevant co-morbidity in HES data using the Charlson index has been reported to underestimate its prevalence and therefore provides suboptimal stratification for the purpose of outcome comparison.

Preoperative risk stratification may be used to help patients weigh up the risks and benefits of surgery as part of the process of informed consent. It can also be used to identify elective and emergency patients who might benefit most from management in a high dependency or intensive care unit setting. A predictive scoring system must be effective and should be demonstrated to improve outcome by modifying patient management. Ease of implementation is also an important consideration. A score that is already routinely collected in many hospitals as part of the admission process would offer a distinct advantage.

The most widely used prospective risk stratification tool is the American Society of Anesthesiologists (ASA) grade, which forms part of the World Health Organization preoperative checklist, mandated in UK National Health Service (NHS) hospitals.^3^ While the benefit of this system is that it is simple, there is significant interobserver variation, making it inadvisable to use on its own to grade surgical risk.^4^ More comprehensive systems include the Physiological and Operative Severity Score for the enUmeration of Mortality and morbidity (POSSUM) and its modifications (the Portsmouth or P-POSSUM), the Simplified Acute Physiology Score II (SAPS II) and the Acute Physiology and Chronic Health Evaluation II (APACHE II) score.^5–7^ The latter systems require the collection of at least 12 variables and are not yet incorporated into routine practice in most hospital settings.

The Waterlow score was developed in the mid-1980s and is used widely in the UK to stratify the risk of decubitus ulcer development among the inpatient population.^8^ It was developed primarily to provide a focus for education, intervention and resource management in the prevention of decubitus ulceration but has been validated subsequently by others.^9^ It is a semiquantitative assessment including factors relating to body mass index, sex, age, tissue perfusion, neurological compromise, extent of surgery, mobility and medications. A numeric score is derived from these variables, which may be used to stratify patients into risk categories (eg Waterlow score >20 suggests a very high risk of decubitus ulcer). In a publication from our institution in 2011, the Waterlow score was found to be elevated in 13 of 16 documented mortalities, leading us to investigate its effectiveness as a surgical risk stratification tool.^10^

The primary aim of this study was to assess the utility of the Waterlow score in predicting mortality and morbidity in a selected retrospective cohort of general surgical and vascular patients at a district general hospital. A secondary aim was to compare the discriminatory power of the Waterlow score with other commonly used systems of perioperative risk stratification. These results would then influence the design of a prospective study measuring the utility of the Waterlow score for real-time risk assessment.

## Methods

A consecutive series of 331 patients undergoing elective or emergency general or vascular surgery over a 5-month period in 2010 was identified through a single institution database. Inclusion criteria consisted of age over 50 years, surgery undertaken in the main theatre suite and surgical severity defined as major or complex major with reference to the AXA PPP healthcare schedule of procedures.^11^ These criteria were chosen to select a moderate to high risk patient group in whom risk assessment could be evaluated. A total of 101 patients matched our criteria and were included in the study.

Two authors (CT and MS) independently extracted the variables required for the calculation of the P-POSSUM for each patient. An online risk calculator provided by the Vascular Anaesthesia Society of Great Britain and Ireland (http://www.vasgbi.com/riskpossum.htm) was employed to calculate these scores. Where a difference in score was found, the mean score was used. The ASA grade was extracted from the anaesthetic chart as documented preoperatively. Preoperative Waterlow scores were extracted from nursing notes with their time of documentation relative to surgery. Current policy at our institution dictates that the Waterlow score is calculated by nursing staff on the day of admission for all surgical patients. A widely adopted revision of the Waterlow score, incorporating a measure of malnutrition, was used in the hospital trust.^12,13^

**Table 1:** Detail of the range of elective and emergency operations

**Elective procedures**	**65**
Cholecystectomy	15
Colorectal resection	14
Vascular bypass/endarterectomy	8
Hernia repair (incisional/recurrent)	6
Varicose vein surgery (recurrent)	6
AAA repair	3
Restoration intestinal continuity	3
Mastectomy	2
Iliofemoral endarterectomy	2
Other	6
**Emergency procedures**	**36**
Emergency laparotomy	18
Vascular bypass/endarterectomy	6
Amputation	5
Appendicectomy	2
Cholecystectomy	2
Gastrojejunostomy	1
AAA repair	1
Strangulated incisional hernia repair	1

AAA = abdominal aortic aneurysm

Postoperative outcome data extracted from case notes included 30-day morbidity and inpatient mortality. Postoperative morbidity was defined as acute renal failure, bleeding requiring ≥4 units of red cell transfusion within 72 hours after surgery, cardiac arrest requiring cardiopulmonary resuscitation, coma for ≥24 hours, deep venous thrombosis, myocardial infarction, unplanned intubation, ventilator use for ≥48 hours, pneumonia, pulmonary embolism, stroke, wound disruption, deep or organ/space surgical site infection, sepsis, septic shock, systemic inflammatory response syndrome and vascular graft failure.^14^ In the event of discharge prior to 30 days, interval outpatient attendances and readmissions were reviewed to determine whether the patient had presented to the hospital with a complication.

Statistical analysis was performed using Prism^®^ 5 (GraphPad, La Jolla, CA, US). Continuous score data were compared using the Mann–Whitney U test for non-parametric data and categorical data were compared using Fisher’s exact test. The discriminatory power of the scoring systems was compared using receiver operating characteristic (ROC) curves, the area under the curve (AUC) and likelihood ratios (LR).

## Results

Over half (55%) of the 101 patients included in the study were female. The median age of the patient group was 68 years (interquartile range: 61–76 years). The median inpatient length of stay (LOS) was 9 days (range: 1–101 days). The inpatient mortality was 17% (17/101), (4/65 elective, 13/36 emergency) and the 30-day morbidity was 29% (28/96).

**Figure 1:**
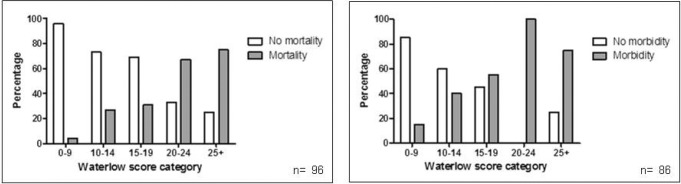
Relative inpatient mortality and 30-day morbidity with reference to the preoperative Waterlow score

**Figure 2:**
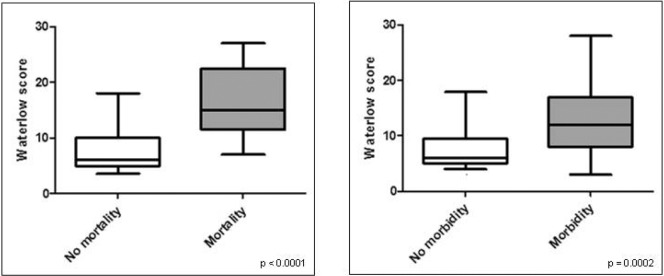
Box plots representing the relationship between preoperative Waterlow score and inpatient mortality and 30-day morbidity (Mann–Whitney U test).

Thirty-three per cent of patients were discharged to level 2 or 3 beds from theatre (19 of 36 emergency cases and 14 of 65 elective cases) and the mean LOS was 1.3 days. The majority of elective operations comprised cholecystectomy (23%), colorectal resection (22%) and vascular procedures (19%). The emergency case mix included emergency laparotomy, colonic resection and acute cholecystectomy (56%), and vascular bypass, endarterectomy or amputation (33%) (Table 1). Compliance with obligatory Waterlow scoring in the preoperative period was 90% and was performed at a median of 2 days before operation (range: 0–17 days) but most commonly on the day of surgery.

**Figure 3:**
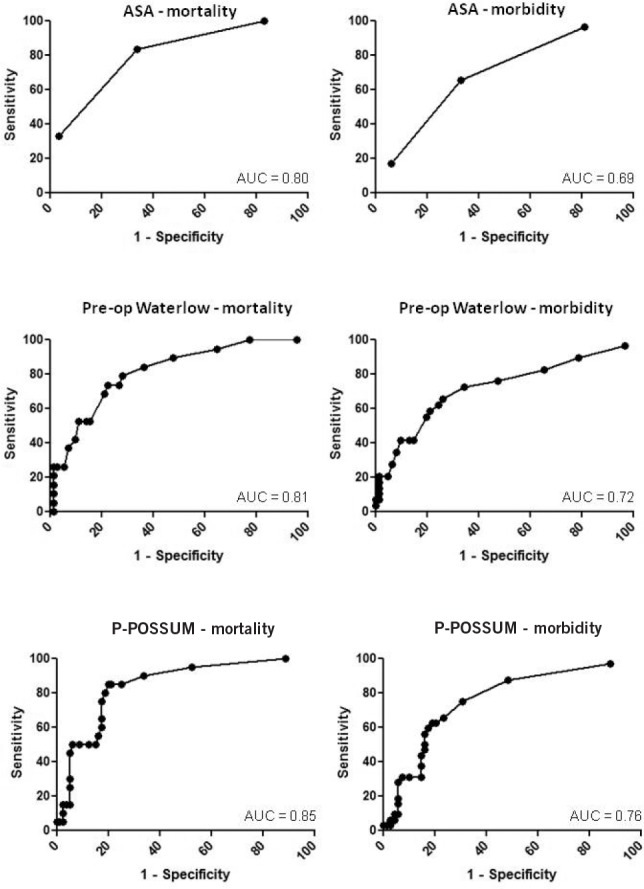
Receiver operating characteristic curves demonstrating the discriminatory power of the scores to predict inpatient mortality and 30-day morbidity

Mortality and morbidity increased with preoperative Waterlow score (Fig 1). A statistically significant association was demonstrated between the preoperative Waterlow score and both mortality (*p*<0.0001) and morbidity (*p*=0.0002) using the Mann–Whitney U test (Fig 2). ROC curve analysis of mortality and morbidity (Fig 3) demonstrated good discrimination using the preoperative Waterlow score (AUC: 0.81, 95% confidence interval [CI]: 0.71–0.92; and AUC: 0.72, 95% CI: 0.60–0.84).

The optimal threshold for discrimination as determined by the maximal likelihood ratios was 22 for mortality (LR: 18.7) and 20 for morbidity (LR: 12.6). Patients are considered to be at very high risk of decubitus ulceration at a Waterlow score of >20 and this threshold was chosen for further analysis. Subsequent dichotomisation into high and low risk groups was found to identify significantly different populations using Fisher’s exact test for mortality (odds ratio [OR]: 14, 95% CI: 2.5–83, *p*=0.0026) and morbidity (OR: 15, 95% CI: 1.7–38, *p*=0.0069). The corresponding likelihood ratios for mortality and morbidity were 9.34 and 12.62 respectively, with accuracy of 0.84 and 0.76 respectively (Table 2).

P-POSSUM scoring was completed retrospectively in all cases and the ASA grade was recorded prospectively in 77% of cases. The baseline characteristics of these subgroups were compared and found to demonstrate no significant differences with respect to age, proportion of emergencies, median hospital LOS, median intensive care unit LOS, inpatient mortality or 30-day morbidity.

**Table 2:** Performance of the preoperative Waterlow score in predicting outcome using a threshold of 20 to classify two groups

Outcome	Likelihood ratio	Accuracy	*p*-value*
Mortality	9.34	0.84	**0.0017**
Morbidity	12.62	0.76	**0.0073**

* Fisher’s exact test

**Table 3:** Comparison of the overall discriminatory ability of selected scoring systems to predict inpatient mortality in the cohort using receiver operating characteristic curves

Scoringsystem	Valid cases	AUC (95% CI)	*p*-value*
ASA grade	77	0.80 (0.69–0.92)	**0.0001**
P-POSSUM	101	0.85 (0.76–0.94)	**<0.0001**
Waterlow (preoperative)	90	0.81 (0.71–0.92)	**<0.0001**

AUC = area under the curve; CI = confidence interval; ASA = American Society of Anesthesiologists; P-POSSUM = Portsmouth Physiological and Operative Severity Score for the enUmeration of Mortality and morbidity

* chi-squared test

The relative discrimination of the scoring systems was compared using their ROC curves with respect to inpatient mortality and 30-day morbidity (Fig 3). Results demonstrate that all the scoring systems were generally good in predicting mortality (AUC: 0.80–0.85, *p*≤0.0001) (Table 3) and morbidity (AUC: 0.69–0.76, *p*≤0.005). Preoperative Waterlow scoring therefore appeared to attain equivalent predictive discrimination to more established scoring systems as determined by ROC analysis.

## Discussion

The Waterlow score was based originally on the results of a prospective observational study conducted in an acute hospital inpatient population of 649 surgical, orthopaedic, medical and elderly care patients.^8^ It was developed primarily to provide a focus for education, intervention and resource management in the prevention of decubitus ulceration. The current iteration of the Waterlow score incorporates a formal assessment of nutritional status.^15^ The prevention of pressure ulceration remains a high priority for NHS trusts and demographic trends suggest that its prevalence will increase, implying that the process will be sustained.^16^ All 21 hospital trusts in NHS London were contacted and 19 reported using the Waterlow score for inpatient risk stratification.

The preoperative Waterlow score was found to be a highly accurate predictor of outcome in this moderate to high risk surgical cohort. It demonstrated an AUC of 0.81, which compared favourably with the P-POSSUM (AUC: 0.85) and ASA grades (AUC: 0.80) with respect to mortality and was similarly positioned when morbidity was assessed. The prevalence (pre-test probability) of mortality in the study cohort was 17% and the likelihood ratio associated with a positive test (preoperative Waterlow >20) was 9.34, corresponding to an estimated post-test probability of 70%.

Inpatient mortality (17%) was chosen as an outcome measure rather than 30-day mortality, as a previous publication has demonstrated that mortality after emergency surgery increases after the 30th postoperative day from 9% to 22% at 1 year in a similar cohort.^17^ A relative excess of mortality was noted in the emergency subgroup compared with the elective subgroup (37% [13/36] vs 6% [4/65]). Inpatient mortalities followed emergency surgery for amputation (*n*=4), lower limb revascularisation (*n*=4), laparotomy (*n*=4) and hernia repair (*n*=1). Elective mortalities occurred following open abdominal aortic aneurysm repair (*n*=1), lower limb amputation (*n*=1) and lower limb revascularisation (*n*=2).

The Waterlow score incorporates the assessment of various patient factors well recognised to influence surgical outcome. These include body mass index, age, nutritional status, organ failure, anaemia, smoking, medical co-morbidity, drug history, and the duration and type of surgery. Other factors such as the patient’s continence, skin integrity and mobility are not typically considered during surgical risk assessment although these factors would empirically be considered to influence outcome. By comparison, the ASA grade classifies the presence and degree of organ failure while the P-POSSUM includes age, operative details and organ failure among its variables.

There was little difference in the performance of the objective, numeric score (P-POSSUM) and the more subjective, semiquantitative ASA grade or Waterlow score. The use of clinical judgement and experience in determining the ASA grade and Waterlow score may in some way compensate for the actuarial precision but inflexibility of strictly quantitative scores such as P-POSSUM. It is not known whether the good performance of the ASA grade in outcome prediction can be replicated without assessment by an anaesthetist.

There are several limitations of this study. Scoring systems such as Waterlow and ASA grade containing subjective or operator dependent variables always carry the risk of reduced inter-rater (and even intra-rater) reliability and agreement.^18,19^ While predictive tools that use more objective parameters try to eliminate this problem, they have serious implementation issues. This was a relatively small sample retrospective study in a selected cohort aimed at initial evaluation of the utility of the Waterlow score in risk stratification. Results should be confirmed in a larger prospective study.

It is not unreasonable to select a high risk cohort since this group might benefit most from stratification and modification of treatment. There was some variation in the timing of the preoperative Waterlow score assessment. It was not our aim to change existing procedures for the purpose of the study but rather to see if the existing system could predict surgical risk with minimal adjustment. The variables that contribute to the score are relatively insensitive to acute physiological changes and it is unlikely the scores would change significantly over the days prior to surgery.

The AUC summary statistic in ROC analysis is useful to compare scoring systems but there is no absolute threshold that demonstrates clinical effectiveness. The likelihood ratio is a summary statistic that can be applied independently of a condition’s prevalence in a study population. A publication from 2005 suggests that a mortality rate representative of normal colorectal surgical practice is 3.4%.^20^ At this prevalence, the post-test probability of mortality in patients with a Waterlow score of >20 can be calculated as approximately 25%. This is clearly lower than a mortality of 70%, calculated from the selected population used in this study, but would nevertheless define a group with a high predicted mortality.

A report by The Royal College of Surgeons of England and the Department of Health working group on perioperative care of the higher risk surgical patient recommended that all patients should have their expected risk of death documented prior to surgery.^21^ In this report, high risk patients are defined as having a predicted hospital mortality of ≥10%. The high prevalence of mortality and morbidity in the emergency surgical population has also been highlighted by the College with similar recommendations.^22^ The advantage of using the Waterlow score is that it is already measured routinely on admission to many hospitals in the UK and could therefore be easily integrated into the surgical admission process. A prospective comparative evaluation of the Waterlow score and ASA grade in surgical outcome prediction in a standardised study protocol is required.
